# IGF2BP1 promotes multiple myeloma with chromosome 1q gain via increasing CDC5L expression in an m^6^A-dependent manner

**DOI:** 10.1016/j.gendis.2024.101214

**Published:** 2024-01-17

**Authors:** Jiadai Xu, Yawen Wang, Liang Ren, Panpan Li, Peng Liu

**Affiliations:** a Department of Hematology, Zhongshan Hospital, Fudan University, Shanghai 200030, China; b Cancer Center, Zhongshan Hospital, Fudan University, Shanghai 200030, China

**Keywords:** CDC5L, Chromosome 1q gain, IGF2BP1, Multiple myeloma, N6-methyladenosine

## Abstract

Multiple myeloma (MM) patients with chromosome 1q gain (1q+) are clinically and biologically heterogeneous. The underlying molecular mechanisms are still under investigation, while the identification of targets for effective therapy of this subgroup of MM patients is urgently needed. We aimed to investigate the clinical significance and the regulatory mechanisms of insulin-like growth factor 2 messenger RNA (mRNA) binding protein 1 (IGF2BP1), a N6-methyladenosine (m^6^A) reader, in MM patients with 1q+. We found that MM patients with 1q+ exhibit a significantly higher level of IGF2BP1 mRNA than controls, while higher IGF2BP1 expression predicted a worse prognosis in MM patients with 1q+. IGF2BP1 overexpression promoted cell proliferation and G1-to-S phase transition of the cell cycle in NCI-H929 cells. Through comprehensive *in silico* analyses of existing public datasets and in-house generated high-throughput sequencing datasets, along with *in vitro* experiments, we identified CDC5L as a target of IGFBP1, which can bind to the m^6^A sites of CDC5L mRNA to up-regulate its protein abundance. Higher CDC5L expression also predicted a worse prognosis of MM patients with 1q+. Moreover, both knockdown and mutation of CDC5L attenuated the pro-proliferative effect of IGF2BP1. Furthermore, IGF2BP1 inhibitor BTYNB effectively inhibited CDC5L expression in MM cells with 1q+ and suppressed the proliferation of these cells *in vitro* and *in vivo*. Therefore, IGF2BP1 acts as a post-transcriptional enhancer of CDC5L in an m^6^A-dependent manner to promote the proliferation of MM cells with 1q+. Our work identified a novel IGF2BP1-CDC5L axis and provided new insight into developing targeted therapeutics for MM patients with 1q+.

## Introduction

Multiple myeloma (MM) is characterized as a malignant overgrowth of abnormal plasma cells,^1^ which remains incurable. Cytogenetic abnormalities always confer inferior outcomes.^2^ Chromosomal 1q copy number gain (1q+) is one of the most common cytogenetic abnormalities in MM.^3^ However, the prognostic value of 1q+ in MM has always been controversial and has not been uniformly adopted as a high-risk cytogenetic abnormality according to world guidelines.^3^ This suggests that the MM patients with 1q+ are clinically and biologically heterogeneous. To better guide clinical management, the identification of more powerful prognostic factors and targets for further stratifying and therapy of this subgroup of patients is urgently needed.

RNA epigenetic modifications play an essential role in RNA-associated gene regulation. N6-methyladenosine (m^6^A) is considered to be the most prevalent modification in higher eukaryotic messenger RNAs (mRNAs).^4^ An increasing number of studies have shown that m^6^A modification may be of great importance for the development of MM.^5^, ^6^, ^7^ Insulin-like growth factor 2 messenger RNA binding proteins (IGF2BPs) are a class of RNA-binding proteins that include IGF2BP1, IGF2BP2, and IGF2BP3, which regulate the localization, translation, or turnover of their target transcripts.^8^ IGF2BPs possess two RNA recognition motifs at the N-terminal and four K-homologous domains at the C-terminal. Distinct K-homologous domains are necessary for binding with various target RNAs. The consequences of point mutations within any of the four K-homologous domains could significantly impact the binding outcomes.^9^ Consequently, despite sharing structural similarities, IGF2BP1-3 display variations in their tissue expression patterns, target mRNA specificity, and functional roles.^10^ In 2018, Huang et al reported that IGF2BPs are a new family of m^6^A readers that stabilize m^6^A-modified mRNAs and alter the translation efficiency of their targets.^11^ Notably, their study highlighted the importance of IGF2BPs as m^6^A readers in post-transcriptional gene regulation in cancer biology. IGF2BP1 is a multifunctional RNA-binding protein that binds to diverse mRNAs to protect the stability of targeted mRNAs by shielding them from degradation while promoting translation by binding to the 3′-untranslated region of several transcripts.^12^ It has been revealed that IGF2BP1 can bind to the coding sequence of several mRNAs, such as c-Myc, β-TrCP1 (BTRC), and PTEN.^13^^,^^14^ Although the role of IGF2BP1 in the progression of diverse tumors has been well documented,^15^^,^^16^ whether and how IGF2BP1 impacts MM developments remains largely unexplored.

In the current study, we found that IGF2BP1 promoted MM cells with 1q+, and a higher expression level of IGFBP1 predicted a worse prognosis in MM patients with 1q+. Cell division cycle 5-like (CDC5L) was identified as a target of IGF2BP1. CDC5L mRNA can be modified by IGF2BP1 in an m^6^A-dependent manner. In addition, this work studied the therapeutic potential of BTYNB, a small molecule functionally inhibiting IGF2BP1,^17^ in suppressing the growth of MM cells with 1q+ in *in vitro* and *in vivo* models.

## Materials and methods

Between December 2017 and December 2020, bone marrow aspirates were collected from 16 patients with monoclonal gammopathy of undetermined significance (MGUS) and 23 patients with newly diagnosed MM (Table 1) at the Department of Hematology, Zhongshan Hospital, Fudan University. This study was approved by the Ethics Committee of Zhongshan Hospital (approval number: B2017-031R), and all included patients gave their written informed consent. The diagnosis for MGUS and MM was established in accordance with the International Myeloma Working Group criteria of 2018^18^ and 2010.^19^ The collection of bone marrow mononuclear cells and sorting of CD138^+^/CD138^−^ plasma cells were performed with the MACS separator and CD138-coated magnetic beads (Miltenyi Biotec, MB17-R0009) as previously described.^20^ The datasets of CoMMpass study Interim Analysis (IA) 15 funded by the Multiple Myeloma Research Foundation (MMRF) were used in this study.

**Table 1:** The clinical baseline of patients whose CD138^+^ plasma cells were isolated for RNA sequencing.

	Monoclonal gammopathy of undetermined significance	Newly diagnosed multiple myeloma
Cases (number)	16	30
Age, mean (range)	63.8 (53–77)	66.5 (48–87)
Sex (%)		
Male	10 (62.5)	20 (66.7)
Female	6 (37.5)	10 (33.3)
del (17p)		
(−)	11 (100.0)	22 (81.5)
(+)	0 (0.0)	5 (18.5%)
NA	5	3
del (13q)		
(−)	11 (100.0)	13 (48.1%)
(+)	0 (0.0)	14 (51.9%)
NA	5	3
1q gain		
(−)	9 (81.8)	9 (33.3)
(+)	2 (18.2)	18 (66.7)
NA	5	3
t (11; 14)		
(−)	8 (72.7)	26 (96.3)
(+)	3 (27.3)	1 (18.5)
NA	5	3
t (4; 14)		
(−)	11 (100.0)	21 (77.8)
(+)	0 (0.0)	6 (22.2)
NA	5	3
t (14; 16)		
(−)	11 (100.0)	26 (96.3)
(+)	0 (0.0)	1 (3.7)
NA	5	3

All human multiple myeloma cell lines (HMCLs) have been authenticated using STR profiling within the last three years. All experiments were performed with mycoplasma-free cells. The below experiment methods were detailed in the supplementary methods, including western blot (WB) and real-time quantitative PCR (RT-qPCR),^20^^,^^21^ cell culture, lentiviral transduction and generation of stable transfection cell lines, cell proliferation and cell cycle analyses,^22^ 5-Ethynyl-2′-deoxyuridine (EdU) incorporation assay, the observation of multinucleation, the analysis of cell apoptosis, RNA extraction, RNA sequencing (RNA-seq),^23^, ^24^, ^25^, ^26^ methylated RNA immunoprecipitation sequencing (MeRIP-seq) and MeRIP-qPCR,^27^ crosslinking immunoprecipitation sequencing (CLIP-seq), RNA co-immunoprecipitation-quantitative PCR,^20^ mRNA stability assay,^28^ *in vivo* xenograft mouse model, survival analysis, and statistical analysis.

## Results

### IGF2BP1 displays a high expression level in MM patients with 1q+ and its high expression predicts poor prognosis in these patients

**Figure 1:**
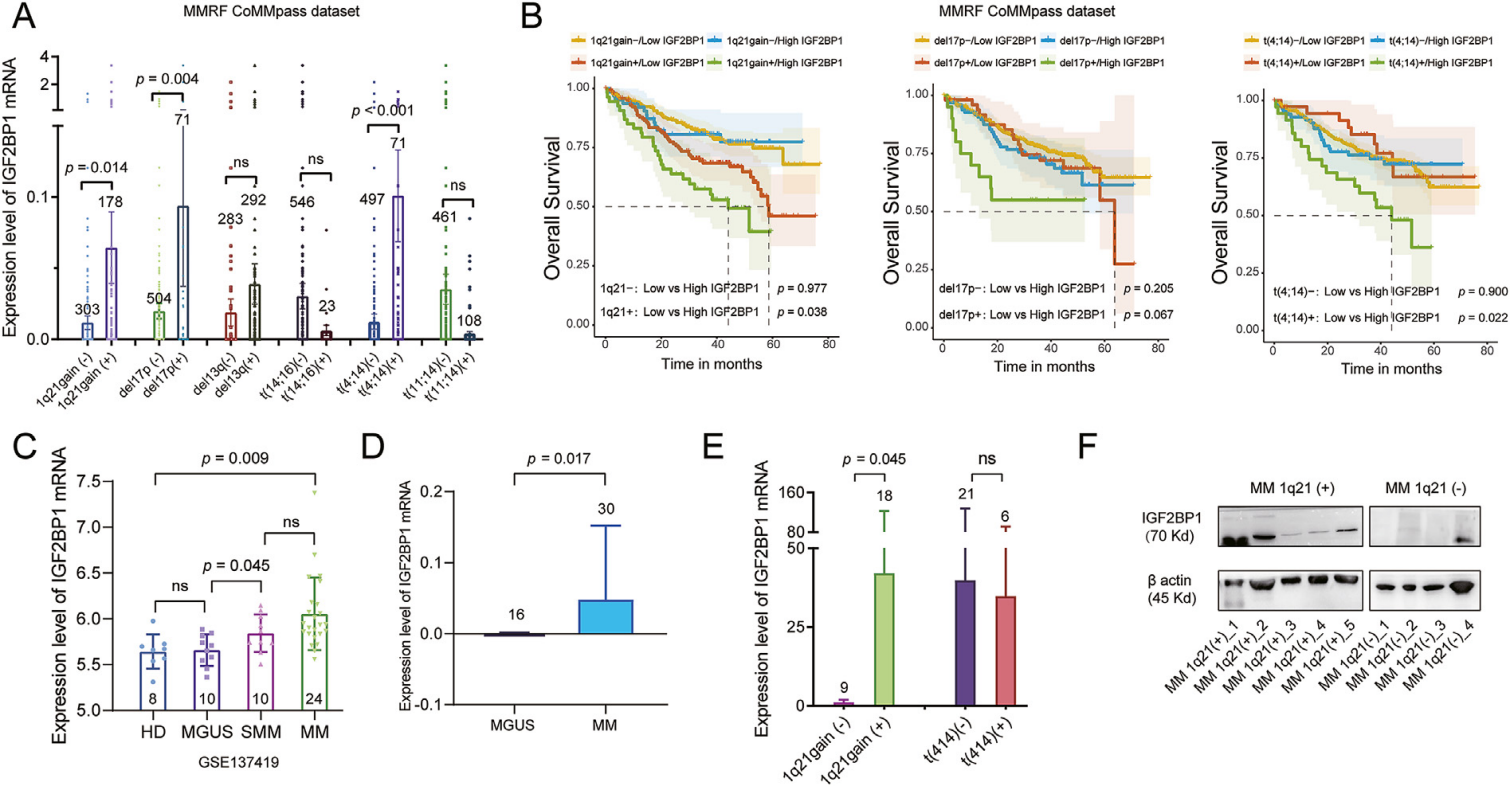
IGF2BP1 displays a high expression level in MM patients with 1q+ and its high expression predicts poor prognosis in these patients. **(A)** In the MMRF CoMMpass dataset, the relationship between IGF2BP1 mRNA levels and six kinds of CAs was quantitated. **(B)** In MM patients with 1q+ or t(4;14), those with a higher IGF2BP1 mRNA expression level exhibited worse OS. The IGF2BP1 mRNA expression levels were not found to be prognostically important in assessing OS in MM patients with del17p. Expression levels of IGF2BP1 mRNA were not prognostically important for assessing OS in MM patients without 1q+, del17p, or t(4;14). **(C)** The GSE24080 dataset was used to compare the expression level between healthy donor, MGUS, SMM, and MM groups. **(D)** The mRNA levels of IGF2BP1 in RNA-seq data of CD138^+^ plasma cells from MM patients and MGUS patients in Zhongshan Hospital were analyzed. **(E)** Validation of IGF2BP1 mRNA expression by qRT-PCR determined that MM patients with 1q+ had significantly higher levels of IGF2BP1 mRNA. **(F)** By western blot assays, CD138^+^ plasma cells from MM patients with 1q+ expressed a higher level of IGF2BP1 protein compared with plasma cells from MM patients without 1q+. IGF2BP1, insulin-like growth factor 2 mRNA binding protein 1; MM, multiple myeloma; OS, overall survival; CAs, cytogenetic abnormalities; SMM, smoldering MM.

To clarify the relationship between IGF2BP1 expression and cytogenetic abnormalities, including 1q+, chromosome 17p deletion (del17p), chromosome 13q deletion (del13q), t(4;16), t(4;14), and t(11;14), we firstly analyzed the RNA-seq data from the MMRF CoMMpass dataset. Notably, MM patients with 1q+ (*n* = 178; *P* = 0.014), del17p (*n* = 71; *P* = 0.004), and t(4;14) (*n* = 71; *P* < 0.001) were found to exhibit significantly higher levels of IGF2BP1 mRNA (Fig. 1A). Based on the medians of IGF2BP1 mRNA expression levels, we evaluated the impacts of IGF2BP1 mRNA on overall survival of MM patients with 1q+, del17p, or t(4;14) by Kaplan–Meier survival analysis (log-rank test). In MM patients with 1q+ or t(4;14) (Fig. 1B), those with a higher IGF2BP1 mRNA expression level were found to have a worse prognosis in terms of overall survival as compared with the patients with a lower IGF2BP1 mRNA level (1q21+: *P* = 0.038, t(4;14)+: *P* = 0.022). The expression level of IGF2BP1 was not found to be prognostically important in assessing overall survival in MM patients with del17p (Fig. 1B; *P* = 0.067). Expression levels of IGF2BP1 mRNA were not prognostically important for assessing overall survival in MM patients without 1q+, del17p, or t(4;14). Next, the GSE24080 dataset was used to compare the IGF2BP1 expression levels between the healthy donor (*n* = 8), MGUS (*n* = 10), smoldering MM (*n* = 10), and MM (*n* = 24) groups. The healthy donor group and MGUS group performed similar expression levels of IGF2BP1. Markedly elevated IGF2BP1 expression levels were found in the smoldering MM group compared with the MGUS group (*P* = 0.045; Fig. 1C). Furthermore, we compared the expression levels of IGF2BP1 in RNA-seq data of CD138^+^ plasma cells from MM patients (*n* = 30) or MGUS patients (*n* = 16) in our department, and observed a significantly higher level of IGF2BP1 mRNA in MM patients (*P* = 0.017; Fig. 1D). By qRT-PCR validation, MM patients with 1q+ had significantly higher levels of IGF2BP1 mRNA (Fig. 1E; *P* = 0.045), while MM patients with t(4;14) failed to reach statistical significance. Finally, by WB assays, we confirmed that CD138^+^ plasma cells from MM patients with 1q+ (*n* = 5) expressed higher levels of IGF2BP1 protein compared with plasma cells from MM patients without 1q+ (*n* = 4) (Fig. 1F).

### IGF2BP1 promotes the proliferation and cell cycle progression of HMCLs

**Figure 2:**
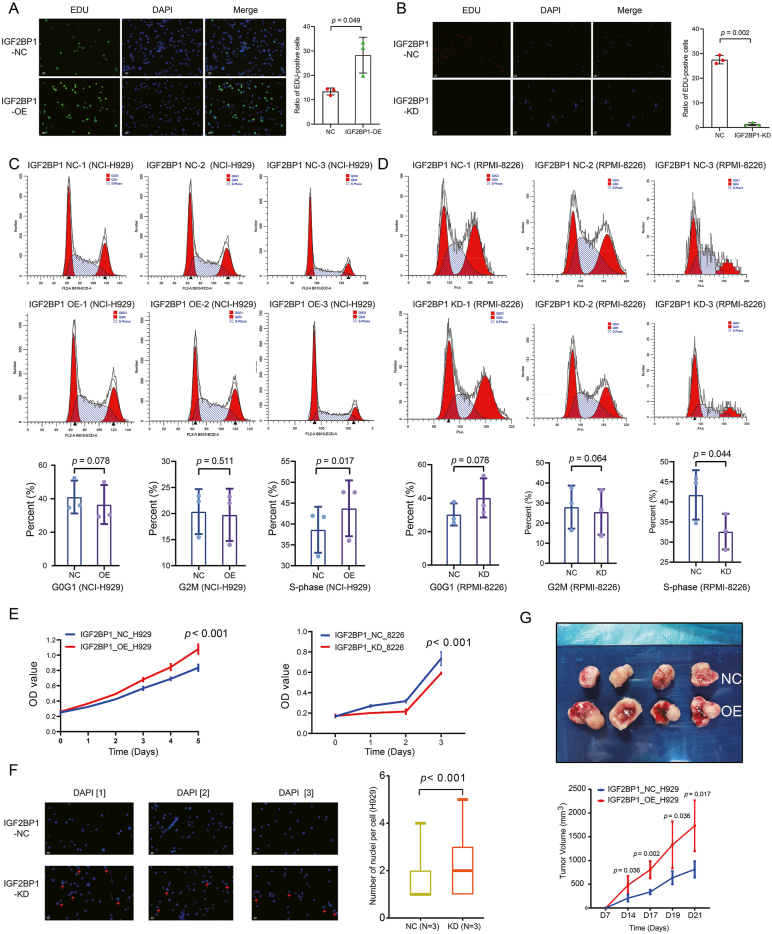
IGF2BP1 promotes the proliferation and cell cycle progression of HMCLs. **(A, B)** In the IGF2BP1-OE group, the ratio of EdU-positive cells was significantly higher. In the IGF2BP1-KD group, the ratio of EdU-positive cells was significantly lower. The percentages of EdU-positive cells were calculated from five randomly selected view regions of each slide. **(C, D)** Overexpression of IGF2BP1 promoted cell cycle progression by increasing the proportion of the S phase. Knockdown of IGF2BP1 inhibited RPMI-RPMI-8226 cell cycle progression by decreasing the proportion of the S phase in the cell cycle. Representative flow profiles of three independent experiments are shown, and the percentages of NCI-H929 cells at G0/G1, G2/M, and S phases were summarized. **(E)** The proliferation of NCI-H929 cells of the IGF2BP1-OE group and RPMI-RPMI-8226 cells of the IGF2BP1-KD group and NC group were determined by CCK8 assays at the indicated culture durations. **(F)** IGF2BP1-KD NCI-H929 cells showed a significantly higher multinucleation rate than the control cells. Representative images of DAPI staining are shown (red arrows indicate multinucleation), and the numbers of nuclei per cell were summarized. **(G)** IGF2BP1-OE NCI-H929 cells resulted in a ∼2.13-fold increase in tumor volume, relative to IGF2BP1-NC cells at day 21 after inoculation into B-NDG mice. IGF2BP1, insulin-like growth factor 2 mRNA binding protein 1; HMCLs, human multiple myeloma cell lines.

**Supplementary Figure 1:**
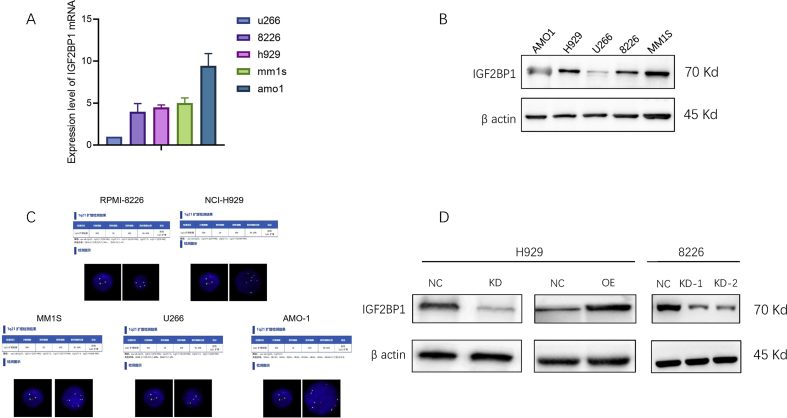
The baseline characteristics of human multiple myeloma cell lines (HMCLs) in this study. **A.** The baseline mRNA level of IGF2BP1 in five kinds of HMCLs, including AMO1, NCI-H929, U266, RPMI-8226 and MM1S. **B.** The baseline protein level of IGF2BP1 in five kinds of HMCLs, including AMO1, NCI-H929, U266, RPMI-8226 and MM1S. **C.** The five kinds of HMCLs were all confirmed to have 1q+ via fluorescence *in situ* hybridization (FISH). **D.** The overexpression and inhibition of IGF2BP1 in NCI-H929 and RPMI-8226 cells were confirmed by western blot assay.

The baseline mRNA and protein expression levels of IGF2BP1 in five kinds of HMCLs, including AMO1 (RRID: CVCL_1806), NCI-H929 (RRID: CVCL_1600), U266 (RRID: CVCL_T031), RPMI-8226 (RRID: CVCL_0014), and MM1.S (RRID: CVCL_8792), were shown in Figure S1A, B. The five kinds of HMCLs were all confirmed to have 1q+ via fluorescence *in situ* hybridization^20^ (Fig. S1C). The transfection cell lines NCI-H929-IGF2BP1-OE, NCI-H929-IGF2BP1-KD, and RPMI-RPMI-8226-IGF2BP1-KD were thus constructed and verified to show effective overexpression and silence of IGF2BP1, respectively, as depicted in Figure S1D. The NCI-H929-IGF2BP1-OE group was evidently more proliferative, as the ratio of EdU-positive cells (green) was found to be significantly higher than the control group (*P* = 0.049; Fig. 2A). The RPMI-8226-IGF2BP1-KD group were evidently less proliferative, as the ratio of EdU-positive cells (red) was found to be significantly lower than the control group (*P* = 0.002; Fig. 2B). The cell cycle analysis with a paired sample *t*-test suggested that the overexpression of IGF2BP1 promoted NCI-H929 cell cycle progression by increasing the proportion of S phase in the cell cycle (repeated three times, S phase: *P* = 0.017; Fig. 2C). Knockdown of IGF2BP1 inhibited RPMI-RPMI-8226 cell cycle progression by decreasing the proportion of S phase in the cell cycle (repeated three times, S phase: *P* = 0.044; Fig. 2D).

Subsequently, CCK-8 assay further supported that overexpression of IGF2BP1 stimulated the proliferation of NCI-H929 (*P* < 0.001) and knockdown of IGF2BP1 suppressed the proliferation of RPMI-RPMI-8226 (*P* < 0.001; Fig. 2E). DAPI staining suggested that NCI-H929-IGF2BP1-KD cells had a significantly higher multinucleation rate than the control cells (*P* < 0.001; Fig. 2F), suggesting the appearance of mis-segregated uncondensed chromosomes,^29^ the cell cycle arrest at the pre-S phase, and induction of cell death.^30^

We further examined the *in vivo* growth differences between IGF2BP1-OE and IGF2BP1-NC cells after subcutaneously inoculating these cells to B-NDG mice. The IGF2BP1-OE NCI-H929 cells (1731.0 ± 535.1 mm^3^) showed a ∼2.13-fold increase in tumor volume compared with the IGF2BP1-NC cells (813.2 ± 171.7 mm^3^) (day 14, *P* = 0.036; day 17, *P* = 0.002; day 19, *P* = 0.036; day 21, *P* = 0.017; Fig. 2G). Taken together, IGF2BP1 promotes the proliferation and cell cycle progression of HMCLs.

### Combinatory analyses of MeRIP-seq, RNA-seq, CLIP-seq, and patients' survival data identified CDC5L mRNA as a potential target of IGF2BP1 in MM

**Figure 3:**
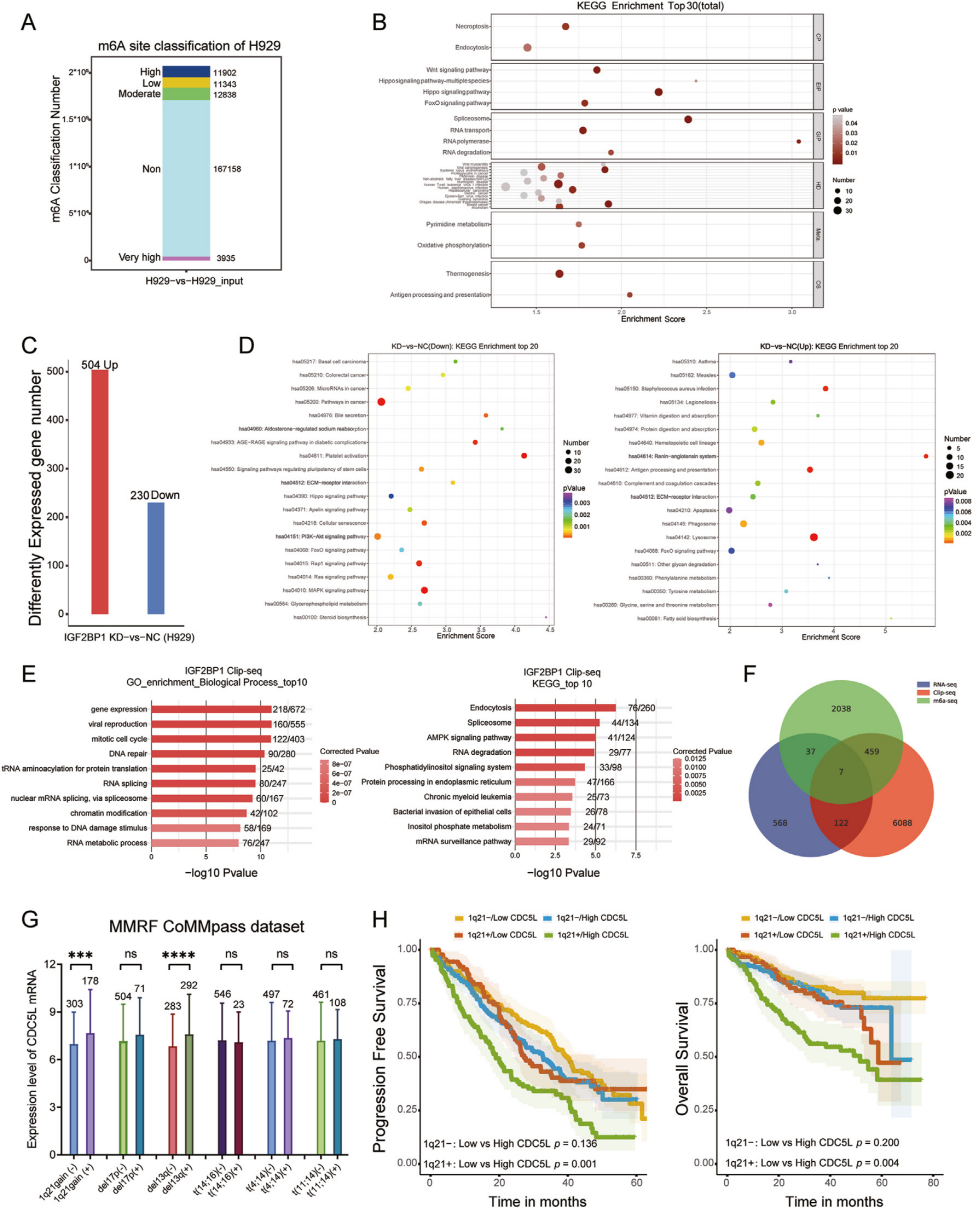
Combinatory analyses of MeRIP-seq, RNA-seq, CLIP-seq, and patients' survival data identified CDC5L mRNA as a potential target of IGF2BP1 in MM. **(A)** The numbers of predicted m^6^A site classification based on reliability in NCI-H929 cells were predicted by the SRAMP tool. **(B)** The top 30 significant enriched KEGG pathways for the 2608 peak-related genes in MeRIP-seq were plotted according to enrichment score and pathways. The *P*-value and number of genes are denoted by color darkness and size of cycle, respectively. **(C, D)** The top 20 obviously enriched KEGG pathways of down-regulated differentially expressed gene (DEGs) (*n* = 230; C) and up-regulated DEGs (*n* = 504; C) in the IGF2BP1-KD RNA-seq data were plotted according to enrichment scores and pathways (D). **(E)** The top 10 significant enriched KEGG pathways of the target genes of IGF2BP1 from Clip-seq data analysis were plotted. **(F)** The overlapping of differentially expressed mRNAs for m^6^A-seq, IGF2BP1-KD RNA-seq, and IGF2BP1 CLIP-seq versus control are shown in the Venn diagram. **(G)** The relationship between CDC5L expression levels and six classical CAs of MM patients in the MMRF dataset. **(H)** In MM patients with 1q+, patients with a higher CDC5L mRNA expression level exhibited worse prognosis in terms of progression-free survival (PFS) and overall survival (OS) as compared with the patients with a lower CDC5L mRNA level. IGF2BP1, insulin-like growth factor 2 mRNA binding protein 1; MM, multiple myeloma; CAs, cytogenetic abnormalities; CDC5L, cell division cycle 5-like; MeRIP-seq, methylated RNA immunoprecipitation sequencing; CLIP-seq, crosslinking immunoprecipitation sequencing; RNA-seq, RNA sequencing.

**Supplementary Figure 2:**
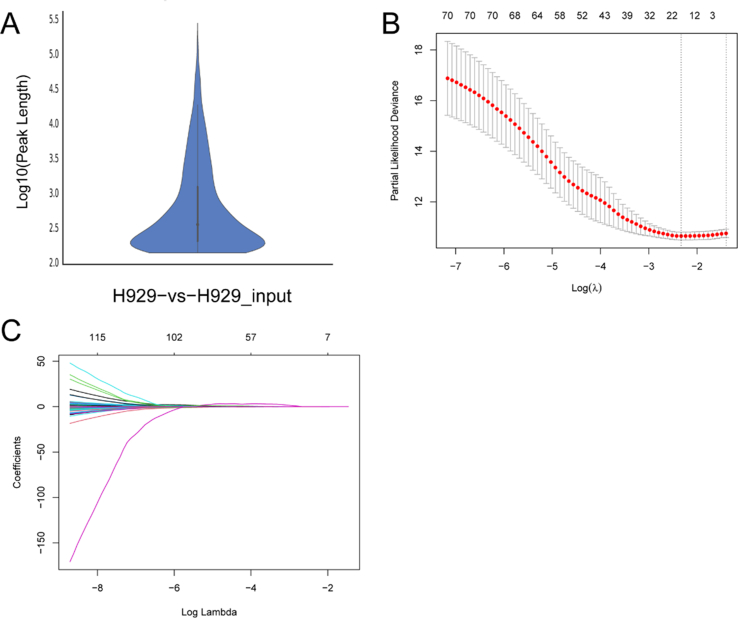
Combinatory analyses of MeRIP-seq, RNA-seq, CLIP-seq and patients' survival data identified CDC5L mRNA as a potential target of IGF2BP1 in MM. **A.** The width of the methylation peak indicates the width of the mRNA sequence where the methylation site exists. A statistical violin plot of the width distribution of the peaks is shown. **B. and C.** In MMRF CoMMpass dataset, 466 genes were used for LASSO regression analysis and multivariant Cox regression with OS as the survival endpoint.

We profiled the transcriptome-wide distribution of RNA m^6^A in NCI-H929 by MeRIP-seq. Here, the width of the methylation peak indicated the width of the mRNA sequence in which the methylation site existed. A statistical violin plot of the width distribution of the peaks is shown in Figure S2A. The sequence-based RNA adenosine methylation site predictor (SRAMP) tool^31^ was used to identify potential m^6^A sites in the above peak. The number of predicted m^6^A site classifications based on reliability in NCI-H929 is shown in Figure 3A. The KEGG analysis resulted in 2608 peak-related genes annotated in 300 KEGG pathways. A scatter plot shows the top 30 significantly enriched KEGG pathways, including spliceosome, Hippo signaling pathway, Wnt signaling pathway, and pathways related to cancers (Fig. 3B).

RNA-seq using IGF2BP1-KD NCI-H929 cells and control cells was conducted. In NCI-H929 IGF2BP1-KD cells, pathways in cancer progression were found to be the most significantly enriched in relation to IGF2BP1 down-regulation (*P* < 0.001, *Q* < 0.001), suggesting that IGF2BP1 might play a significant role in MM development (Fig. 3C, D).

When IGF2BP1 acts as an m^6^A reader, it is a post-transcriptional or protein translational regulator. The Clip-seq using anti-IGF2BP1 antibody in NCI-H929 cells led to the identification of 6676 peak-associated genes. Significant GO enrichment of biological processes pertaining to the mitotic cell cycle, DNA repair, and RNA splicing were noted. The top 10 significantly enriched KEGG pathways of the target genes of IGF2BP1 were shown to be endocytosis, spliceosome, and the AMPK signaling pathway (Fig. 3E).

Next, a Venn diagram was used to illustrate the overlap of differentially expressed genes/mRNAs resulted from m^6^A-seq, IGF2BP1-KD RNA-seq, and IGF2BP1 CLIP-seq analyses (Fig. 3F). As IGF2BP1 is an m^6^A reader, overlapping peak-associated genes of IGF2BP1 Clip-seq and MeRIP-seq (466 genes) were screened. Since the 466 mRNAs were occupied by IGF2BP1 proteins and modified by m^6^A simultaneously, we chose them for further survival analysis. The forest map of genes associated with overall survival by multivariant Cox regression analysis was presented in Figure S2B. With *P* < 0.05 as the screening condition, eight prognosis-related genes were identified for overall survival in MM patients with 1q+ from the MMRF dataset (GNB1L, E2F1, CDC5L, NR2F6, LINC00963, VPS25, PEX3, CPQ) (Table 2). To prevent overfitting of the model, LASSO regression analysis was used to test these genes and determined that there was no overfitting for these genes (Fig. S2C). The hazard ratio value greater than 1 indicates that the exposure factor is the promoter of the positive event (such as death). Therefore, independent poor-risk factors affecting the overall survival of MM patients with 1q+ were found to be GNB1L (*P* < 0.001; hazard ratio: 1.2; 95% confidential interval: 1.1–1.3), CDC5L (*P* = 0.009; hazard ratio: 1.1; 95% confidential interval: 1–1.2), and E2F1 (*P* = 0.018; hazard ratio: 1.1; 95% confidential interval: 1–1.1). Since CDC5L protein was demonstrated to act as a positive regulator of cell cycle G2/M progression,^32^ we compared the relationship between CDC5L expression levels and six classical cytogenetic abnormalities and evaluated the impacts of CDC5L expression on survival of MM patients in the MMRF dataset. Notably, MM patients with 1q+ (*P* = 0.002) and 13q deletion (*P* < 0.001) were found to exhibit significantly higher levels of CDC5L mRNA (Fig. 3G). In MM patients with 1q+, those with a higher CDC5L mRNA expression level exhibited a worse prognosis in terms of progression-free survival (*P* = 0.001) and overall survival (*P* = 0.004) as compared with patients with a lower CDC5L mRNA level. Expression levels of IGF2BP1 mRNA were not prognostically important for assessing overall survival in MM patients without 1q+ (Fig. 3H). These results were in line with the observation that IGF2BP1 promoted the proliferation and cell cycle of NCI-H929 cells. Therefore, further analyses were carried out to elucidate the relationship between IGF2BP1 and CDC5L.

**Table 2:** Multivariant Cox regression for overall survival in multiple myeloma patients with 1q+ from MMRF CoMMpass dataset.

Gene	*P* value	Hazard ratio (95% confidential interval)
PEX3	0.044	0.92 (0.84–1)
CDC5L	0.0091	1.1 (1–1.2)
E2F1	0.018	1.1 (1–1.1)
CPQ	0.026	0.98 (0.96–1)
VPS25	0.017	1 (1–1.1)
NR2F6	0.024	0.92 (0.86–0.99)
GNB1L	0.00026	1.2 (1.1–1.3)
LINC00963	0.0053	1 (1–1.1)

### CDC5L is a target gene of IGF2BP1 through mRNA modification in an m^6^A-dependent manner in HMCLs with 1q+

**Figure 4:**
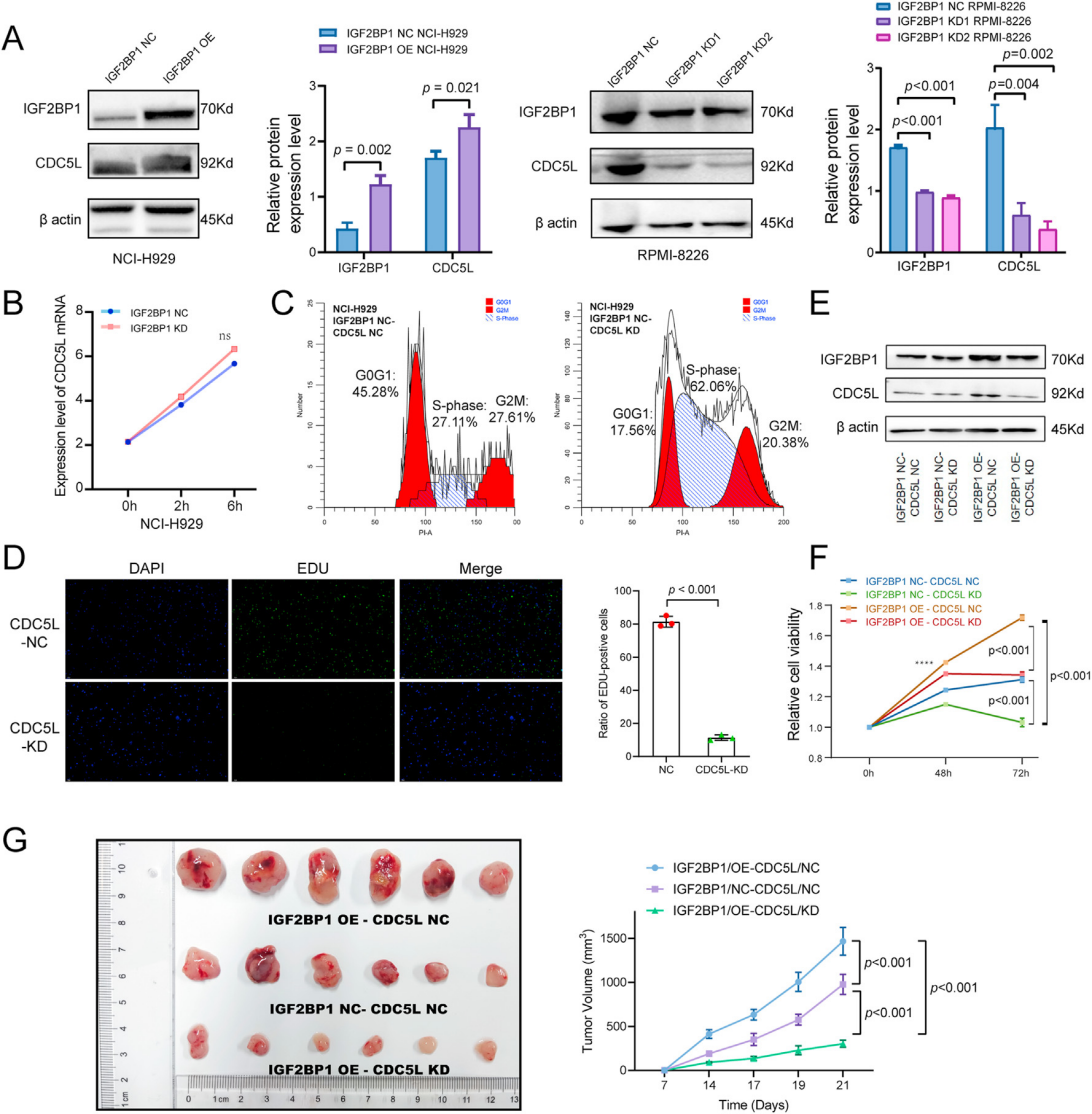
CDC5L mediates the proliferative effect of IGF2BP1 in MM cells. **(A)** Compared with NCI-H929-IGF2BP1-NC cells, NCI-H929-IGF2BP1-OE cells had increased expression of CDC5L protein, as shown by western blot results. Compared with RPMI-RPMI-8226-IGF2BP1-NC cells, RPMI-RPMI-8226-IGF2BP1-KD cells had less expression of CDC5L protein. **(B)** IGF2BP1 expression did not affect the stability of CDC5L mRNA. NCI-H929 cells were treated with actinomycin D at the indicated time durations, and CDC5L mRNA levels were quantitated by quantitative PCR. **(C)** Cell cycle assays showed that knockdown of CDC5L would result in S-phase arrest. **(D)** The ratio of EdU-positive cells (green) in the RPMI-RPMI-8226-CDC5L-KD group was found to be significantly lower than in the control group. **(E)** An IGF2BP1/OE-CDC5L/KD rescue cell line was constructed. **(F)** Knockdown of CDC5L attenuated the proliferative effect of IGF2BP1 on NCI-H929 cells by CCK8 assays (∗∗∗*P* < 0.001). **(G)***In vivo*, we assessed the growth disparities among IGF2BP1/NC-CDC5L/NC, IGF2BP1/OE-CDC5L/NC, and IGF2BP1/OE-CDC5L/KD cells after subcutaneously inoculating these cells to B-NDG mice. CDC5L, cell division cycle 5-like; IGF2BP1, insulin-like growth factor 2 mRNA binding protein 1; MM, multiple myeloma.

Compared with NCI-H929-IGF2BP1-NC cells, NCI-H929-IGF2BP1-OE cells had more expression of CDC5L protein (*P* = 0.021). Compared with RPMI-8226-IGF2BP1-NC cells, RPMI-8226-IGF2BP1-KD cells had significantly less expression of CDC5L protein (KD1: *P* = 0.004, KD2: *P* = 0.002) (Fig. 4A). Next, the mRNA stability assays using transcription inhibition actinomycin D at the indicated time durations indicated that IGF2BP1 did not affect the stability of CDC5L mRNA (Fig. 4B), and the increase in CDC5L protein level may not be due to the increase in stability of mRNA. Next, the transfection cell line NCI-H929-CDC5L-KD was constructed. Results of cell cycle assay suggested that knockdown of CDC5L would result in S-phase arrest (from 27.11% to 62.06%), which was in accordance with previous studies^33^ (Fig. 4C). EdU assay was then performed to analyze the difference in cell proliferation ability between the RPMI-8226-CDC5L-KD group and the CDC5L-NC group. The RPMI-8226-CDC5L-KD group was evidently less proliferative, as the ratio of EdU-positive cells (green) was found to be significantly lower than the control group (*P* < 0.001; Fig. 4D). Additionally, to confirm if CDC5L mediates the proliferative effect of IGF2BP1 in NCI-H929 cells, an IGF2BP1/OE-CDC5L/KD rescue cell line was constructed (Fig. 4E). As shown in Figure 4F, knockdown of CDC5L attenuated the proliferative effect of IGF2BP1 overexpression in NCI-H929 cells (*P* < 0.001). *In vivo*, we further assessed the growth disparities among IGF2BP1/NC-CDC5L/NC, IGF2BP1/OE-CDC5L/NC, and IGF2BP1/OE-CDC5L/KD cells after subcutaneously inoculating these cells to B-NDG mice. Notably, the IGF2BP1/NC-CDC5L/KD cells exhibited a significantly sluggish growth rate *in vitro*, consequently rendering them non-tumorigenic *in vivo*. On day 21, the tumor volume of IGF2BP1/OE-CDC5L/KD NCI-H929 cells (302.18 ± 39.80 mm^3^) was significantly smaller than IGF2BP1/OE-CDC5L/NC cells (1465.70 ± 157.47 mm^3^; *P* < 0.001) and IGF2BP1/NC-CDC5L/NC cells (976.33 ± 114.00 mm^3^; *P* < 0.001) (Fig. 4G). These findings indicate that the effect of IGF2BP1 on the proliferation of NCI-H929 cells might be mediated by CDC5L.

**Figure 5:**
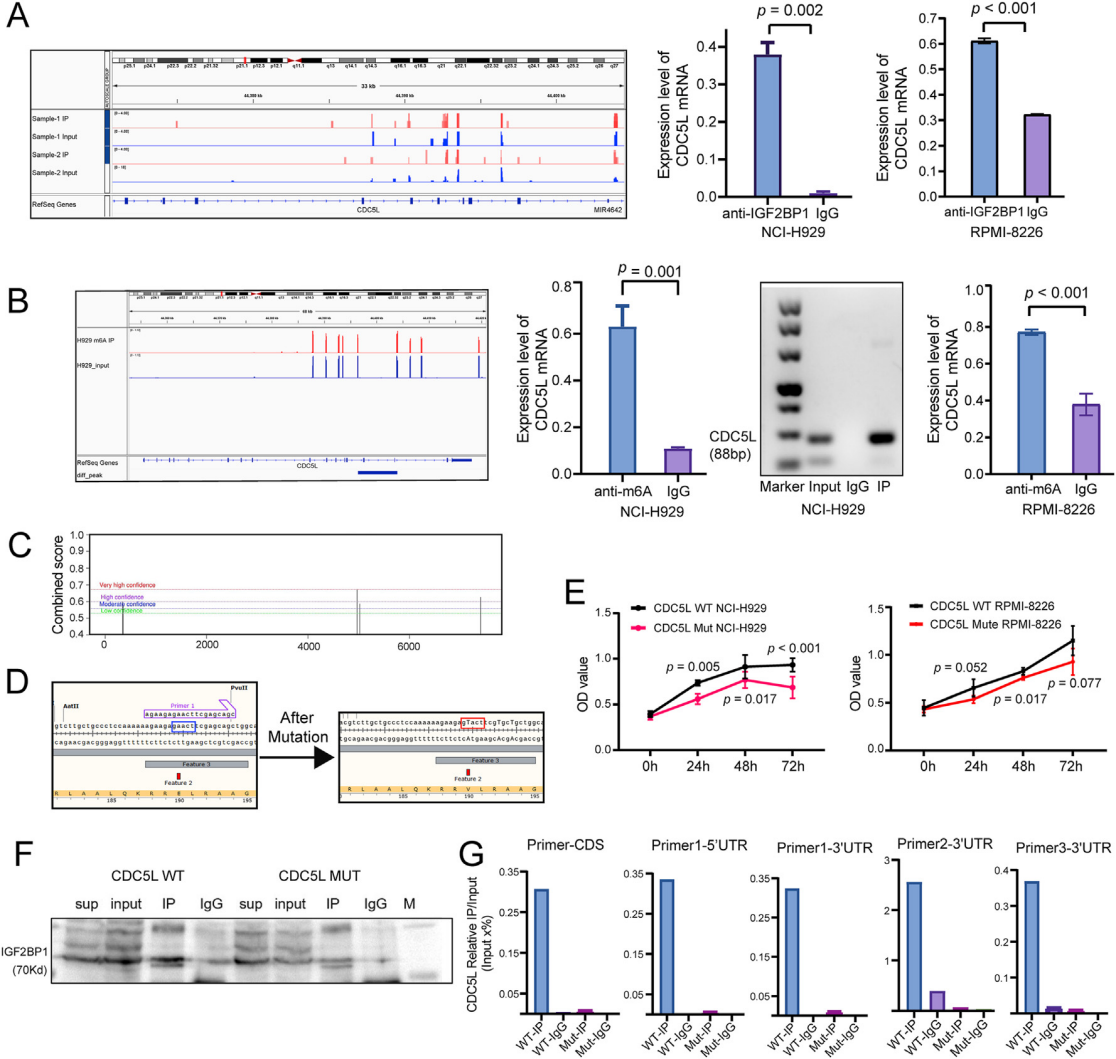
CDC5L is a target gene of IGF2BP1 through mRNA modification in an m^6^A-dependent manner in HMCLs with 1q+. **(A)** The BAM files of CDC5L mRNA produced by IGF2BP1 Clip-seq were visualized by the Integrative Genomics Viewer (IGV). The PCR products of the IP sample in agarose gel slices were confirmed to contain IGF2BP1 and CDC5L by quantitative PCR and western blot assays. **(B)** The IGV tracks displaying the MeRIP-seq read distribution in CDC5L mRNA of NCI-H929 cells. Real-time quantitative PCR of MeRIP products was performed to verify the m^6^A modification sites on CDC5L mRNA. **(C)** m^6^A modification sites of CDC5L mRNA predicted by SRAMP database. **(D)** 44403838 on CDC5L coding sequence (blue) was selected as the m^6^A mutation site (red) to remove m^6^A modification. **(E)** The proliferation rate of CDC5L-MUT cells and CDC5L-WT cells were measured by CCK-8 assays at the indicated time points (NCI-H929, RPMI-RPMI-8226). **(F)** The interaction between CDC5L-WT/CDC5L-MUT and IGF2BP1 in NCI-H929 cells was detected using co-immunoprecipitation-quantitative PCR assays, while the IP efficiency was assessed by western blot assays (sup means supernatant). **(G)** IGF2BP1 exhibited an enriched binding with CDC5L-WT mRNA, but a considerably reduced binding with CDC5L-mut RNA. The percentages of CDC5L relative IP/input were quantitated by quantitative PCR using different primer pairs among the indicated four groups. IGF2BP1, insulin-like growth factor 2 mRNA binding protein 1; HMCLs, human multiple myeloma cell lines; CDC5L, cell division cycle 5-like; MeRIP-seq, methylated RNA immunoprecipitation sequencing; CLIP-seq, crosslinking immunoprecipitation sequencing.

Based on the above results, to specify the correlation between IGF2BP1 protein and CDC5L mRNA, the Bam files of CDC5L mRNA produced by IGF2BP1 Clip-seq (two replicates, IP-1 *vs*. Input-1, IP-2 *vs*. Input-2) were firstly visualized using the Integrative Genomics Viewer (IGV). The blue shading corresponds to the RNA-seq mapping distribution of input, while the red signal corresponds to IGF2BP1 Clip-seq peaks on CDC5L mRNA. The Y-axis in the bar graph was reads per million mapped reads, representing the abundance of reads. After verification with qRT-PCR (*P* = 0.002) for the immunoprecipitation samples of NCI-H929 (*P* = 0.002) and RPMI-RPMI-8226 (*P* < 0.001) (anti-IGF2BP1 *vs*. IgG), the positive interaction between IGF2BP1 protein and CDC5L mRNA was confirmed (Fig. 5A).

IGV tracks were shown to display MeRIP-seq read distribution in the CDC5L mRNA from NCI-H929 cells. The blue shading corresponds to the total RNA-seq mapping distribution (input), while the red signal corresponds to MeRIP-seq peaks on CDC5L mRNA (Fig. 5B). RT-qPCR of the MeRIP products was then performed to verify the m^6^A modification sites on CDC5L mRNA in NCI-H929 cells and RPMI-RPMI-8226 cells (Fig. 5B). Meanwhile, the SRAMP database was employed to predict the m^6^A modification sites of CDC5L mRNA, including one very high confidence site, two high confidence sites, and two moderate confidence sites (Fig. 5C and Table 3). Considering the combinational sites between CDC5L and IGF2BP1 resulting from the Clip-seq peaks data, 44403838 (high confidence) was chosen as the mutation site (gaact to gUact) to remove the m^6^A modification (Fig. 5D). The target binding sites of shRNA were “AGAAGAGAACTTCGAGCAGC”. This site was consistent with the “RRACH” consensus motif (R, purine; H, non-guanine base), the most common m^6^A methylation modification site.

**Table 3:** m^6^A site of CDC5L mRNA predicted by SRAMP dataset.

Chromosome	Start	End	A position	Score (binary)	Score (spectrum)	Score (combined)	m^6^A site classification
Chr6	44396438	44403957	44401405	0.692	0.649	0.675	Very high confidence
Chr6	44396438	44403957	44403838	0.657	0.586	0.628	High confidence
Chr6	44396438	44403957	44396783	0.693	0.461	0.6	High confidence
Chr6	44396438	44403957	44401457	0.626	0.525	0.586	Moderate confidence
Chr6	44396438	44403957	44396792	0.61	0.521	0.574	Moderate confidence

To confirm if IGF2BP1 regulated CDC5L expression through mRNA modification in an m^6^A-dependent manner in NCI-H929 cells, a CDC5L-m^6^A mutation (MUT) cell line was constructed. Compared with the CDC5L-wild type (WT) cell line, the CDC5L-m^6^A mutation (MUT) cell line had a significantly decreased proliferation rate (72 h; *P* < 0.01; Fig. 5E). Furthermore, the interaction between CDC5L-WT/CDC5L-MUT and IGF2BP1 in NCI-H929 cells was detected using a co-immunoprecipitation-quantitative PCR assay, and a good IP efficiency was achieved in both groups (Fig. 5F). Additionally, IGF2BP1 exhibited an enriched binding with CDC5L-WT mRNA, while it had considerably reduced binding with CDC5L-MUT mRNA (Fig. 5G).

**Supplementary Figure 3:**
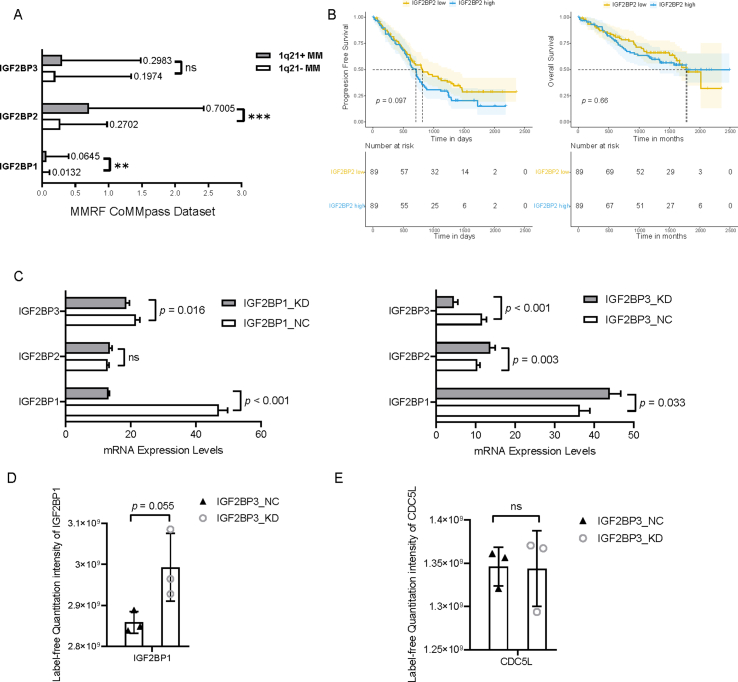
IGF2BP1-3 do not possess redundant or overlapping functions on the CDC5L protein in 1q+ MM. **A.** IG2BP1 and IGF2BP2 both exhibits significantly higher expression in 1q+ MM patients from the MMRF CoMMpass dataset. **B.** IGF2BP2 does not possess prognostic implications for progression-free survival (PFS) (*p* = 0.097) and overall survival (OS) (*p* = 0.66) in 1q+ MM patients. **C.** A notable reduction in IGF2BP3 mRNA expression level (*p* = 0.016) was observed in IGF2BP1-KD NCI-H929 cells, while IGF2BP2 exhibited minimal alteration. The IGF2BP1 (*p* = 0.033) and IG2BP2 (*p* = 0.003) mRNA expression level both increased significantly in IGF2BP3-KD NCI-H929 cells. **D.** The reduction in IGF2BP3 protein level has a marginal effect the increase of IG2BP1 protein level (*p* = 0.055). **E.** The reduction in IGF2BP3 protein level does not have any discernible impact on the protein abundance of CDC5L.

Finally, we combined the MMRF CoMMpass dataset, RNA-seq of IGF2BP1-KD NCI-H929 in comparison to control cells, RNA-seq of IGF2BP3-KD NCI-H929 versus control cells, and label-free quantitative proteomics analysis comparing IGF2BP3-KD NCI-H929 cells to control cells to investigate the potential redundant functions of IGF2BP1, IGF2BP2, and IGF2BP3 on CDC5L. Our findings indicate that IGF2BP1-3 does not possess redundant or overlapping functions on the CDC5L protein in 1q+ MM (Fig. S3).

Collectively, our results suggest that CDC5L mRNA serves as a target of the m^6^A reader protein IGF2BP1, which recognizes and binds to the m^6^A sites to up-regulate the protein abundance of CDC5L.

### The IGF2BP1 inhibitor BTYNB effectively down-regulates CDC5L expression and suppresses the proliferation of MM cells *in vivo*

**Figure 6:**
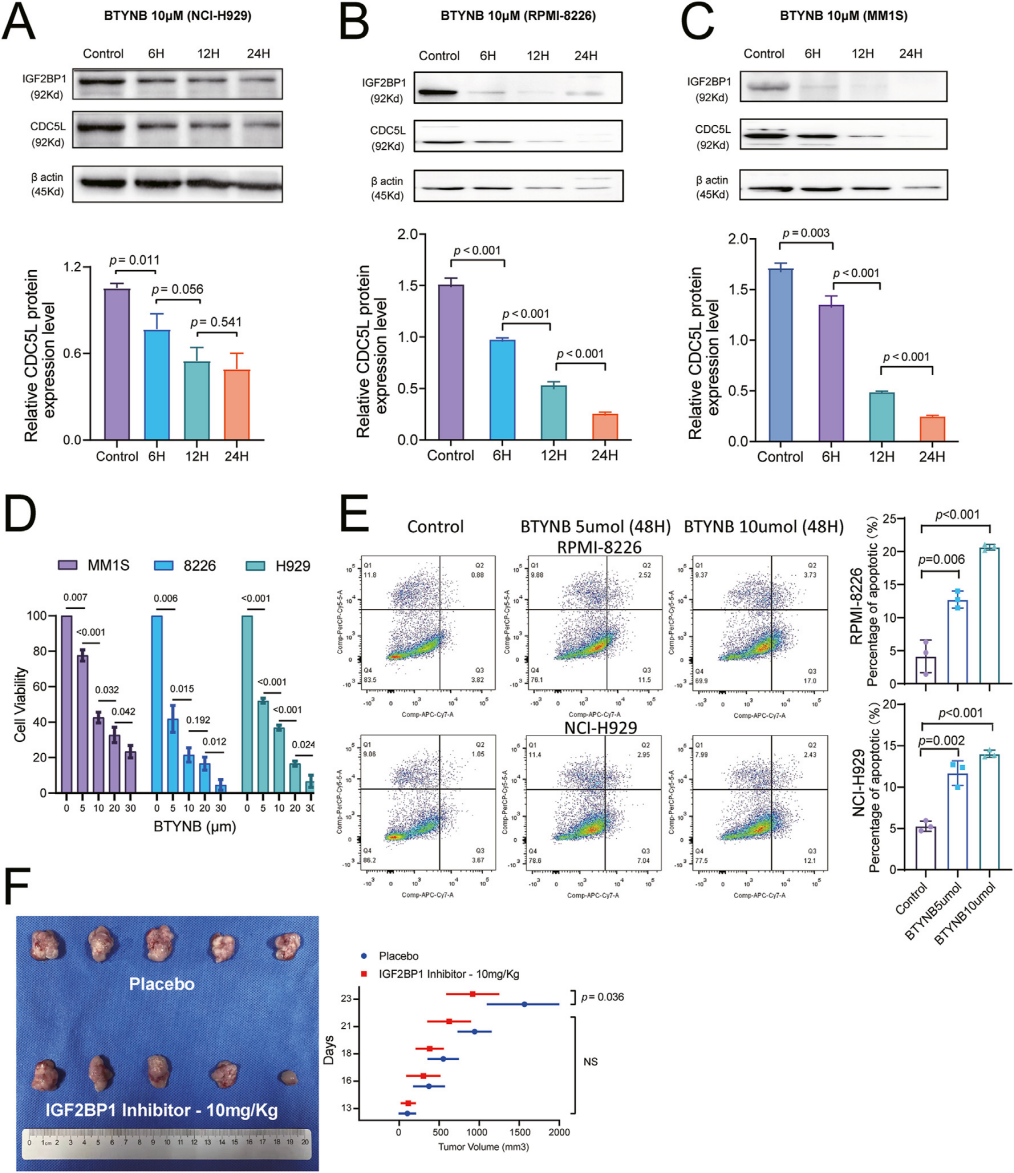
The IGF2BP1 inhibitor BTYNB effectively down-regulates CDC5L expression and suppresses the proliferation of MM cells *in vivo*. **(A–C)** The level of CDC5L protein decreased gradually with the duration of BTY treatment in NCI-H929, RPMI-RPMI-RPMI-8226, and MM1.S cells. **(D)** BTYNB dose-dependently decreased the cell viability of NCI-H929, RPMI-RPMI-RPMI-8226, and MM1.S cells, as revealed by CCK-8 assays of the indicated cells after treatment with BTYNB at the concentrations from 0 to 30 μM for 48 h. **(E)** Flow cytometry analysis was subsequently performed to assess the apoptosis rate of NCI-H929 and RMPI-8226 cells after 48-h exposure to the IGF2BP1 inhibitor BTYNB. The percentage of apoptotic cells, encompassing both early and late apoptosis, exhibited a noteworthy increase with escalating concentrations of BTYNB. **(F)** The B-NDG mice inoculated with NCI-H929 cells were treated with BTYNB or placebo. The images of tumors on day 23 are shown. IGF2BP1, insulin-like growth factor 2 mRNA binding protein 1; CDC5L, cell division cycle 5-like; MM, multiple myeloma.

We further evaluated the therapeutic potential of the IGF2BP1 inhibitor, BTYNB, in treating MM both *in vitro* and *in vivo*. NCI-H929, RPMI-RPMI-8226, and MM1.S cells displayed gradually decreased expression levels of CDC5L along with the increase of BTYNB treatment time during the 24-h treatment period (Fig. 6A–C). As the concentration of BTYNB increased from 0 to 30 μm, the cell viability of NCI-H929, RPMI-RPMI-RPMI-8226, and MM1.S cells gradually decreased (Fig. 6D). Flow cytometry analysis was subsequently performed to assess the apoptosis rate of NCI-H929 and RMPI-8226 cells after a 48-h exposure to the IGF2BP1 inhibitor, BTYNB. The percentage of apoptotic cells, encompassing both early and late apoptosis, exhibited a noteworthy increase with escalating concentrations of BTYNB (Fig. 6E).

To investigate the biological function of BTYNB for MM cells with 1q+ *in vivo*, B-NDG mice were subcutaneously injected with NCI-H929 cells. BTYNB or placebo was then administrated continuously once per day after the subcutaneous tumors were established.

Notably, volumes of the tumors in the BTYNB group were found to be markedly smaller than those in the placebo group on day 23 (*P* = 0.036; Fig. 6F), demonstrating that BTYNB effectively inhibited the growth of MM cells in the xenograft mouse model. Therefore, IGF2BP1 inhibition is a promising approach to treat MM with 1q+.

**Supplementary Figure 4:**
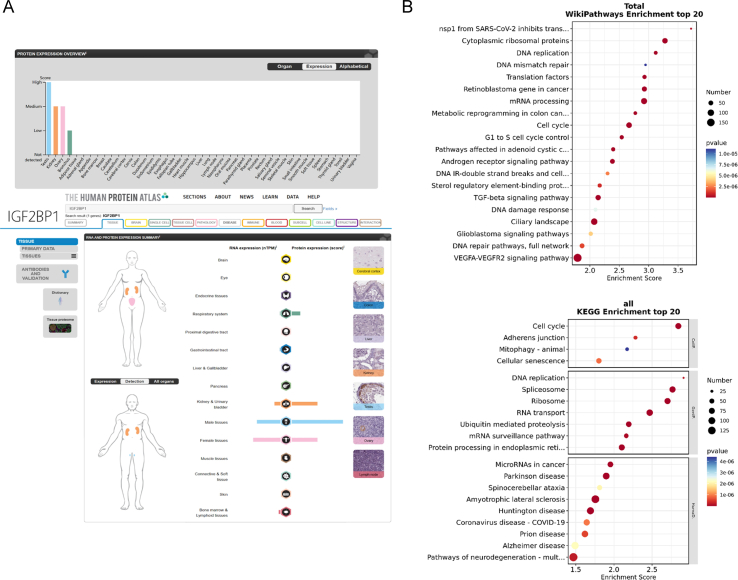
As IGF2BP1 regulates thousands of transcripts, BTYNB may have potential off-target effects. **A.** IGF2BP1 protein expression level in normal tissue from the data of Human Protein Atlas (HPA) database. **B.** WikiPathways and KEGG enrichment analysis on the target genes of IGF2BP1.

As IGF2BP1 regulates thousands of transcripts, BTYNB may have potential off-target effects. We searched the Human Protein Atlas database (https://www.proteinatlas.org/) and identified that the testis, kidney, ovary, and bronchus exhibited higher levels of IGF2BP1 protein expression compared with other normal tissues (Fig. S4A). Subsequently, based on the findings of Huang et al,^11^ we performed WikiPathways and KEGG enrichment analysis on the target genes of IGF2BP1. The results indicated that these target genes primarily participate in DNA repair pathways, DNA replication, RNA splicing, and the cell cycle, among others (Fig. S4B). Hence, it is postulated that the potential off-target consequences of BTYNB might encompass the impairment of reproductive capacity, renal function impairment such as acute necrosis of renal tubular epithelium, and pulmonary toxicity such as interstitial pneumonia and pulmonary fibrosis.

## Discussion

IGF2BP proteins, which are m^6^A readers, help prevent the degradation of their targeted m^6^A-modified mRNAs, such as c-Myc mRNA, in different cancers.^11^^,^^34^, ^35^, ^36^ However, studies pertaining to the role of IGF2BPs in MM are lacking. Our previous study demonstrated that IGF2BP3 binds to CKS1B mRNA located at chromosome 1q21 and promotes tumor proliferation in MM cells with 1q+.Alessandro Canella et al reported that IGF2BP3 binds directly to CD44 mRNA and increases its stability to promote the resistance of lenalidomide and dexamethasone in MM.^37^ Here, with a focus on IGFBP1, we demonstrated for the first time that IGF2BP1 was up-regulated in MM patients with 1q+ and its high expression level could predict a worse prognosis in this subgroup of patients. Functionally and mechanically, through a combination of high-throughput assays, including RNA-seq, Clip-seq, and MeRip-seq, we found that IGF2BP1 promoted the proliferation of MM cells *in vitro* and *in vivo* by acting as a post-transcriptional enhancer of the CDC5L protein in an m^6^A-dependent manner in MM cells with 1q+.

The significant up-regulation of IGF2BP1 in 1q+ MM may be due to the overexpression of certain genes resulting from the copy number gain located at chromosome 1q. HNRNPU protein and DHX9 protein were reported to colocalize with IGF2BP1 in the cytoplasm, and notably, they were found to be associated with IGF2BP1 in a coding region instability determinant-dependent manner, which is essential in ensuring the stabilization of c-Myc mRNA.^38^ The IFI16 protein contributes to B-cell differentiation, which was shown to serve as a prognostic factor for many types of cancers and is involved in immune invasion.^39^^,^^40^ Increased expression of IFI16 predicts an adverse prognosis of MM.^41^ Various cancers are associated with KIF14 overexpression, including pancreatic adenocarcinoma^42^ and ovarian cancer.^43^ KIF14 binds to microtubules and chromatin to form the bipolar spindle, which may be oncogenic.^44^^,^^45^ For MM, expression of KIT14 appears to be bone marrow niche dependent.^46^ These publications may help explain the worse prognosis of high expression levels of IGF2BP1 only in MM patients with 1q+ but not the total MM cases.

The specific role of IGF2BP1 in promoting the cell cycle progression (G to S phase) has been previously reported to involve E2F-driven gene expression as well as other positive regulators of G1/S transition, such as CDK2/4/6 and CCNE1.^34^ CDC5L has been recognized as a positive regulator of G(2)/M progression through transcriptional activation.^32^ Studies have shown that CDC5L is a critical cell cycle mediator for the G2/M transition and mitotic entry.^47^ Tumor cells lacking CDC5L may suffer from mitotic arrest and DNA damage.^48^ The expression of CDC5L in bladder cancer was shown to be obviously increased, and this increase is positively correlated with the pathology grade and the Ki67 expression level.^49^ Liu et al have previously shown that CDC5L could be up-regulated by annexin A7 to promote the cell cycle progression, proliferation, and drug resistance of MM cells.^50^ These reports are consistent with our findings that knockdown of CDC5L significantly reduced the viability of MM cells regardless of endogenous or overexpression of IGF2BP1.

We found that IGF2BP1 promoted the proliferation and cell cycle progression of 1q+ MM cells via modification of CDC5L mRNA in an m^6^A-dependent manner. IGF2BP1 did not affect the stability of CDC5L mRNA, though it up-regulated the protein expression level of CDC5L, suggesting that the post-transcriptional modification of CDC5L mRNA might contribute to the up-regulated protein expression. In addition, CDC5L interference weakened the pro-proliferative effects of IGF2BP1 OE in MM cells, but the viability was not down-regulated to an extent similar to that observed in IGF2BP NC cells, suggesting that CDC5L might be just one of the downstream targets of IGF2BP1, and other possible targets could also contribute to maintaining the viability of MM cells. CDC5L is part of a large multiprotein complex that incorporates into the spliceosome in an ATP-dependent step and participates in DNA repair.^51^ Consistent with the known functions of these proteins, a significant increase in multinucleation rate was observed in NCI-H929-IGF2BP1-KD cells after staining with DAPI. Compared with other forms of nuclear atypia, multiple nucleated cells are prone to displaying a substantially higher incidence of DNA damage.^52^, ^53^, ^54^ However, how IGF2BP1 precisely affects the level of CDC5L protein has not yet been examined in detail, which awaits to be addressed in our future studies.

A previous study identified BTYNB as a potent and selective inhibitor of IGF2BP1 binding to c-Myc mRNA and effectively inhibited the proliferation of IGF2BP1-expressing ovarian cancer and melanoma cells with no effect in IGF2BP1-negative cells.^17^ It has been reported that BTYNB decreases the association of IGF2BP1 with MYC RNA and E2F-driven RNA while inhibiting the proliferation of several tumor cells.^34^ Consistent with our findings in MM cells with 1q+, the proliferation of neuroblastoma cells can also be inhibited by either knocking down IGF2BP1 expression or treatment with the inhibitor BTYNB.^55^ For the first time, we showed that BTYNB suppressed CDC5L expression in MM cells with 1q+ and effectively inhibited their proliferation both *in vitro* and *in vivo*, which implies the candidacy of BTYNB for further therapeutic development. A recent report revealed that BTYNB can act synergistically with the EZH2-inhibitor DZNep in inducing apoptosis of neuroendocrine neoplasms.^56^ Therefore, BTYNB can be used as a reagent to target multiple pathways (C-Myc, CDC5L, EZH2, *etc*.) or be used in combinatorial targeting strategies to modulate the cell cycle and enhance apoptosis of cancer cells more effectively.

One limitation of the current study is its relatively small sample size of MM patients. In addition, all the HMCLs available in our laboratory presented with 1q+. Validation experiments using HMCLs without 1q+ should be done in the future.

In conclusion, we demonstrated that IGF2BP1 modified CDC5L mRNA in an m^6^A-dependent way to promote the proliferation of MM cells with 1q+ and provide novel preclinical evidence of targeting the IGF2BP1/CDC5L axis for effective control of MM development. Our exploration of the therapeutic potential of BTYNB offers a promising strategy for the therapy of MM with 1q+, which warrants further validation in clinical studies.

## Ethics declaration

The research protocol was approved by the Institutional Reviewer Board of The Human Ethics Committee of Zhongshan Hospital, Fudan University (B2017-031R). Written informed consent was obtained from individual or guardian participants. All animal experiments were conducted according to the guidelines of the Animal Care and Use Committee of Fudan University.

## Author contributions

JX performed research, interpreted data, and wrote the paper; YW, LR, and PL did the experimental verification and helped perform the analysis with constructive discussions; PL checked the data and revised the manuscript.

## Conflict of interests

The authors have no conflict of interests.

## Funding

This work was supported by the 10.13039/501100001809National Natural Science Foundation of China (No. 82100215), Natural Science Foundation of Shanghai, China (No. 22ZR1411400), and Natural Science Foundation of Fujian Province, China (No. 2023J05291).

## Data availability

The gene expression datasets generated in this study are available in the GEO database repository (https://www.ncbi.nlm.nih.gov/geo/, GEO accession ID: GSE202975).
