# Supplementary material for: IGF2BP1 promotes multiple myeloma with chromosome 1q gain via increasing CDC5L expression in an m6A-dependent manner
Source: Genes Dis. 2024 Jan 17;12(1):101214. doi: 10.1016/j.gendis.2024.101214 (PMC11554607; doi:10.1016/j.gendis.2024.101214)
Supplement: Multimedia component 1 [file mmc1.docx]

#### Supplementary Methods

**Western blot (WB) and Real-time quantitative PCR (RT-qPCR)**

WB, RNA extraction with TRIzol™ Reagent (Thermo Fisher Scientific, Waltham, MA, USA) and RT-qPCR were performed according to the protocols detailed in our previous studies 1,2. Each experiment was repeated at least three times, and the representative results from one of the repeats are shown. For WB, the primary antibodies were as follows: anti-IGF2BP1 (Novus, NBP1-79024, 1:1000 dilution), anti-CDC5L (Abcam, EPR7658, 1:1000 dilution) and anti-β-actin (Proteintech, 600004-1-lg, 1:2000 dilution). The secondary horseradish peroxidase (HRP)- conjugated antibodies were as follows: goat anti-mouse IgG H&L (Abcam, ab205719, 1: 2000 dilution), goat anti-rabbit IgG H&L (Abcam, ab205718, 1: 2000 dilution). Image quantification was performed using Image J software (National Institutes of Health, Bethesda, MD, USA). For qPCR, the primers were synthesized by Generay Biotechnology (Shanghai, China) and their sequences are listed in **Supplementary Table 1**. Experiments were performed in triplicate. The relative expression level was calculated using the 2−ΔΔCT method.

#### Cell culture

Three human MM cell lines (HMCLs) including NCI-H929, RPMI-8226 and MM1S were purchased from the American Type Culture Collection (ATCC, Manassas, VA, USA). Cell line identity was confirmed by STR-profiling. All cells were cultured in RPMI-1640 medium (HyClone, SH30809.01) containing 10% fetal bovine serum (FBS;

Thermo Fisher Scientific, 10099141) in the incubators with an environment of 37- degree Celsius and 5% CO2.

#### Lentiviral transduction and generation of stable transfection cell lines

The vector for IGF2BP1 overexpression (OE) lentivirus was constructed using a GV341 vector (Ubi-MCS-3FLAG-SV40-puromycin). The vector backbone of IGF2BP1 knock down (KD) shRNA was ‘GV112, hU6-MCS-CMV-Puromycin’, while the target sequences of IGF2BP1 KD shRNA were 5’- CAGGGAAGAATCTATGGCAAA-3’ and 5’-ACAGTAGAGAACTGTGAGCAA-

3’. In addition, the vector backbone of CDC5L knock down (KD) shRNA was ‘GV654, hU6-MCS-Ubiquitin-mCherry-IRES-Neomycin’, while the target sequence of CDC5L KD shRNA was 5’-tgCCAAGACCATCAGAAGTAA-3’. The vectors for CDC5L m6A-mutatioin (MUT) OE lentivirus and CDC5L wild-type (WT) OE lentivirus were constructed using the CV557 vector (Ubi-MCS-3FLAG-SV40-Cherry-IRES- neomycin). The lentiviral vectors construction and lentivirus packaging were conducted by Genechem Co. Ltd, Shanghai, China. NCI-H929, 8226 cells were transduced with lentivirus at a multiplicity of infection of 5-10, and the stable transfectants were selected with puromycin or neomycin.

#### Cell proliferation, cell cycle and apoptosis analyses

Cell proliferation analysis was conducted using a cell counting kit-8 (CCK-8) (Dojindo, Kumamoto, Japan) according to the manufacturer’s instructions 3. The Cell Cycle Assay Kit-PI/RNase Staining (Dojindo, Kumamoto, Japan) was used for cell cycle analysis. Briefly, the harvested cells were collected and fixated in cold 70% ethyl alcohol and stained with propidium iodide (PI) in 1×working solution. Afterward, the cells were distinguished by a BD FACSCalibur™ flow cytometer (BD Biosciences, San Diego, CA, USA) following the producer’s instructions. For the analysis of apoptosis, treated cells were stained with 5μL of APC‐conjugated Annexin V (BD Biosciences, San Diego, CA, USA) for 15 minutes and Cy5-conjugated propidium iodide (PI) (BD Biosciences, No. 51-66211E) for 5 minutes. The samples were analyzed by flow cytometry. Flow cytometric data were analyzed using Flowjo software.

#### 5-Ethynyl-2'-deoxyuridine (EdU) incorporation assay, and observation of multinucleation

Monolayers of cells were prepared by cyto-spinning the cells onto microscope slides. EdU staining was performed using the EdU kit (RiboBio, Guangzhou, China) according to the manufacturer’s instructions. The slides were examined under an Olympus BX53 microscope (Olympus, Shinjuku City, Tokyo, Japan) with the excitation/emission wavelengths of 450–480nm/515nm (green light) and 330–385 nm / 420 nm (ultraviolet light). The ratio of EdU incorporation was calculated as: EdU incorporation (%) = EdU-positive cells (green) / 4',6- diamidino-2-phenylindole (DAPI)-positive cells (blue) × 100. The percentage of EdU-positive cells were calculated from five randomly selected view regions of each slide. DAPI staining was used to observe the multinucleation. Five randomly selected view regions from each slide were used to calculate.

#### RNA extraction, library construction, and RNA-sequencing (RNA-seq)

Total RNA from HMCLs or patients-derived CD138+ PCs was extracted with the EZ- press RNA Purification Kit (Yize Biotechnology Co., Ltd., Shanghai, China) for RNA purification following the manufacturer's instructions. NanoDrop 2000 spectrophotometer (Thermo Scientific) was then used to quantitate the RNA purity. Agilent 2100 Bioanalyzer (Agilent Technologies, Santa Clara, CA, USA) was used to assess the RNA integrity. The libraries were then constructed using VAHTS Stranded mRNA-seq Library Prep Kit for Illumina V2 according to the manufacturer’s protocol. The Illumina sequencing platform (HiSeqTM 2500 or Illumina HiSeq X Ten) sequenced the corresponding libraries. The RNA-seq and data analyses were conducted by OE Biotech Co., Ltd. (Shanghai, China). Raw data (raw reads) of fastq format were initially processed using Trimmomatic 4, after which the low-quality reads (Q20 > 99%, Q30 > 99.9%) were removed. Clean reads were then retained and mapped to the human genome (GRCh38) using HISAT2 5. Fragments Per Kilobase of transcript per Million mapped reads (FPKM) 6 of each gene was calculated using Cufflinks, and the read counts were obtained by HTSeq-count 7.

#### Methylated RNA immunoprecipitation sequence (MeRIP-seq) and MeRIP-qPCR

Total RNA was isolated from NCI-H929 cells using TRIzol reagent. After the RNA was fragmented using fragmentation buffer (10 mM ZnCl2, 10 mM Tris-HCl pH7.0), it was incubated with anti-m6A antibody (Synaptic Systems, 202 003) and was coupled with Dynabeads Antibody Coupling Kit at room temperature while rotating (tail-over- head) at 7 rotations per minute for 1 hour. The samples were then placed on a magnet, which enabled the bead complexes to cluster until the solution became clear. The supernatant was subsequently discarded as it represented the m6A negative fragments that were not captured by the anti-m6A antibody. The m6A-positive mRNA was then eluted from the beads in the elution buffer (0.02 M DTT, 0.150 M NaCl, 0.05 M Tris- HCl pH7.5, 0.001M EDTA, 0.10% SDS, and supplemented with RNase inhibitor at the manufacturer recommended concentration). After extraction and cleanup of the RIP, the m6a-RIP libraries were constructed. The libraries were subjected to single-end sequencing on an Illumina HiSeq 4000 system. For verification, the input RNA and eluted poly(A) RNA were reverse transcribed, and its abundance was tested by real- time PCR. The relative abundance between the input and IP product was calculated by the 2−ΔΔCT method. MeTDiff software 8 (*p* ≤ 0.05; fold change ≥ 1.5) was used to complete the peak calling analysis, while the related genes of the peaks were screened out.

#### Crosslinking immunoprecipitation sequence (CLIP-seq)

#### Crosslinking of NCI-H929 cells was done on ice with UV irradiation type C (254 nm) at 400 mJ per cm2 and cold phosphate buffered saline (PBS). Cells were lysed in cold wash buffer [50mM Tris 7.4, 150mM NaCl, 2mM EDTA, 0.1% SDS, 0.5% NP-40, and 0.5% deoxycholate; supplemented with a 1% RNase inhibitor (Takara) and 2% protease inhibitor cocktail (Roche)] for 30 min. Next, RQ I (Promega, M6101) was added to the lysate to reach a final concentration of 1 U/μL and the lysate was incubated in a 37℃- water bath for 30 min. The stop solution was added to the lysate to quench DNase. The mixture was centrifuged at 13,000 × g at 4℃ for 20 min to remove cell debris.

For immunoprecipitation, the supernatant was incubated overnight at 4℃ with IGF2BP1-antibody (Novus, NBP1-79024) and control IgG-antibody (Sigma, I5006). The protein A /G Dynabeads (Thermo Scientific, 26162) were applied, which was followed by magnet application to remove the supernatants. The beads were sequentially washed with lysis buffer, high-salt buffer (250 mM Tris 7.4, 750 mM NaCl, 10 mM EDTA, 0.1% SDS, 0.5% NP-40 and 0.5 deoxycholate), and PNK buffer (50 mM Tris, 20 mM EGTA and 0.5% NP-40) for two times, respectively. The beads were then resuspended in Elution buffer (50 nM Tris 8.0, 10 mM EDTA and 1% SDS), after which the suspension was incubated for 20 min in a heat block at 70 ℃ to release the immunoprecipitated RNA binding proteins with crosslinked RNA. The immunoprecipitated protein-RNA complex was resolved on a Novex 4-12% Bis-Tris precast polyacrylamide gel (Invitrogen). The protein-RNA complexes were then cut from the gel and RNA was extracted with Trizol after digesting the proteins (Proteinase K, Sangon Biotech, B600169). The cDNA libraries were prepared using the KAPA RNA Hyper Prep Kit (KAPA, KK8541) according the manufacturer’s procedure. For high-throughput sequencing, the libraries were prepared following the manufacturer's instructions and were applied to Illumina NovaSeq 6000 system for 150 nt paired-end sequencing.

#### RNA co-Immunoprecipitation-quantitative PCR (RIP - qPCR)

RIP assays were performed according to the protocol described in our previous study 2. The calculation followed the following formula: Percent Input = 5%×2 ^ (CT Input Sample -CT IP or IgG Sample).

#### mRNA Stability Assay

#### The mRNA stability assay was performed according to the procedure described previously 9. Briefly, NCI-H929 cells transfected with CDC5L-WT or CDC5L-MUT plasmid were seeded into a 6-well plate with the same number of cells in each well. After 24 hours, 5 µg/mL actinomycin D (Act-D) was added to cell culture at 0, 2, and 6 hours prior to collection. The total RNA purification, cDNA synthesis and RT-qPCR were performed as described as above.

***In vivo* xenograft mouse model**

The B-NDG mice (NOD.CB17-PrkdcscidIl2rgtm1/Bcgen, all male, 6 weeks old) were purchased from Beijing Biocytogen Co. (Beijing, China). Mice were firstly subcutaneously injected with NCI-H929 cells (n = 4) and NCI-H929-IGF2BP1-OE (n = 4) (7.5×106 cells per mice). The tumor volume in the two groups were analyzed. After the MM xenograft subcutaneous implanted tumors were formed, an IGF2BP1 inhibitor (BTYNB, MCE, HY-124447; dissolved in corn oil) (n = 5) was given orally to mice at a dosage of 10 mg/Kg body weight, which was based on the results of initial pilot experiments (5mg/Kg, 10mg/Kg, 40mg/Kg). The placebo group (n = 5) received the same dose of corn oil. Tumor size was measured once per week, and tumor volume was calculated by using the following formula: Tumor volume = (Length × Width2)/2. At the end time-point after euthanizing the mice, tumor burden was quantitated by tumor weight and estimated tumor size. All animal experiments were conducted according to the guidelines of Fudan University's Animal Care and Use Committee.

#### Survival analysis and LASSO regression

R packages survival (version 3.5.1) was used for the univariate cox regression analysis, in which “*P* < 0.05” was considered to be statistically significant. To minimize the risk of over-fitting, the least absolute shrinkage and selection operator (LASSO) algorithm was applied for variable selection and shrinkage with the "glmnet" R package. Then, multi-variate cox regression analysis was conducted to identify the independent prognostic biomarkers. Kaplan–Meier (KM) survival curves were generated to compare the prognostic results between different subgroups.

#### Statistical analysis

R (version 3.6.0), SPSS 23.0 and GraphPad Prism 8 were used for generating figures. The consecutive variables were analyzed via Student’s *t*-test. Paired comparisons were performed using a paired *t*-test. All statistical tests were two-sided. A *p* < 0.05 was defined to be statistically significant.

**References**

1. Liu, P. *et al.* Dysregulation of TNFalpha-induced necroptotic signaling in chronic lymphocytic leukemia: suppression of CYLD gene by LEF1. *Leukemia* **26**, 1293-1300, doi:10.1038/leu.2011.357 (2012).
2. Xu, J. *et al.* Globular C1q Receptor (gC1qR/p32/HABP1) Suppresses the Tumor-Inhibiting Role of C1q and Promotes Tumor Proliferation in 1q21-Amplified Multiple Myeloma. *Front Immunol* **11**, 1292, doi:10.3389/fimmu.2020.01292 (2020).
3. Sun, Y. *et al.* Chidamide, a novel histone deacetylase inhibitor, inhibits multiple myeloma cells proliferation through succinate dehydrogenase subunit A. *Am J Cancer Res* **9**, 574- 584 (2019).
4. Bolger, A. M., Lohse, M. & Usadel, B. Trimmomatic: a flexible trimmer for Illumina sequence data. *Bioinformatics* **30**, 2114-2120, doi:10.1093/bioinformatics/btu170 (2014).
5. Kim, D., Langmead, B. & Salzberg, S. L. HISAT: a fast spliced aligner with low memory requirements. *Nat Methods* **12**, 357-360, doi:10.1038/nmeth.3317 (2015).
6. Roberts, A., Trapnell, C., Donaghey, J., Rinn, J. L. & Pachter, L. Improving RNA-Seq expression estimates by correcting for fragment bias. *Genome Biol* **12**, R22, doi:10.1186/gb-2011-12-3-r22 (2011).
7. Trapnell, C. *et al.* Transcript assembly and quantification by RNA-Seq reveals unannotated transcripts and isoform switching during cell differentiation. *Nat Biotechnol* **28**, 511-515, doi:10.1038/nbt.1621 (2010).
8. Cui, X. *et al.* MeTDiff: A Novel Differential RNA Methylation Analysis for MeRIP-Seq Data. *IEEE/ACM Trans Comput Biol Bioinform* **15**, 526-534, doi:10.1109/TCBB.2015.2403355 (2018).
9. Wang, X. *et al.* N6-methyladenosine-dependent regulation of messenger RNA stability.

*Nature* **505**, 117-120, doi:10.1038/nature12730 (2014).

| **Supplementary Table 1.** The sequences of primers used for regular RT-qPCR and RIP-PCR in this study. | | | |
| --- | --- | --- | --- |
| Name | Purpose/Location | Forward (5’ -3’) | Reverse (5’ -3’) |
| IGF2BP1 | qPCR/mRNA | CAGGAGATGGTGCAGGTGTTTATCC | GTTTGCCATAGATTCTTCCCTGAGC |
| CDC5L | qPCR/mRNA | AAGGCCCAGGATGTTTTGGTG | CCTGGTTATAAGCTTCACTTGAGA |
| β actin | qPCR/mRNA | AAGGAGCCCCACGAGAAAAAT | ACCGAACTTGCATTGATTCCAG |
| Primer-CDS | RIP-PCR/CDS | GAAGCCCAGAACCTCATG | TTGTCGCTGTGGAGTTAC |
| Primer1-5’UTR | RIP-PCR/5UTR | CCATTCTGTTTTGGACATGC | AGATATTGGGTGGCTGAAAGG |
| Primer1-3’UTR | RIP-PCR/3UTR | GTTACAGTATCAGTTGGCTAC | CTCTAAGACTCTGGCACAG |
| Primer2-3’UTR | RIP-PCR/3UTR | TCATGGAGGTGACATTAGTG | GCAGTGGTATAGTGGTGAG |
| Primer3-3’UTR | RIP-PCR/3UTR | CCTCAGCCTTCCAAGTAG | TCACACCTGTAATTCTAGCA |
